# Non-Exosomal and Exosomal Circulatory MicroRNAs: Which Are More Valid as Biomarkers?

**DOI:** 10.3389/fphar.2019.01500

**Published:** 2020-01-20

**Authors:** Nik Nur Syazana Binti Nik Mohamed Kamal, Wan Nazatul Shima Shahidan

**Affiliations:** Craniofacial Science Laboratory, School of Dental Sciences, Universiti Sains Malaysia, Health Campus, Kubang Kerian, Malaysia

**Keywords:** exosome, miRNA, biomarker, body fluid, non-exosomal

## Abstract

MicroRNAs (miRNAs) are a group of small non-coding RNAs with approximately 19–25 nucleotides that are involved in regulating a range of developmental and physiological processes. Non-exosomal circulating and exosomal miRNAs have also been proposed to be useful in diagnostics as biomarkers for diseases and different types of cancer. In this review, the quantity of miRNAs and of reliable experimental data analyses of miRNAs that come from exosomal and non-exosomal sources are discussed from the perspective of their use as biomarkers for cancer and other diseases, including viral infections, nervous system disorders, cardiovascular disorders, and diabetes. We summarize other research findings regarding the use of miRNA from these two sources as biomarkers in diagnostics and clinical use. The challenges in using miRNA from these two sources in cancer and disease diagnostics are evaluated and discussed. Validation of specific miRNA signatures as biomarkers is a critical milestone in diagnostics.

## Methodology and Delimitation

A PubMed database search on 4^th^ April 2018 with the keywords “miRNA AND circulating” resulted in a total of 3558 articles. When the parameter “full-text” was applied, 3510 articles remained. After a second parameter, “free full-text,” was applied, a total of 2055 articles were left. These articles were then manually classified into a group of original articles and a group of “others” (not related to the topic, reviews, reports, editorials, commentaries, etc.) Here we are focusing on original articles considering exosomal and non-exosomal circulatory miRNAs as potential biomarkers. A total of 69 original articles were found to be related to the topic.

**Figure 1:**
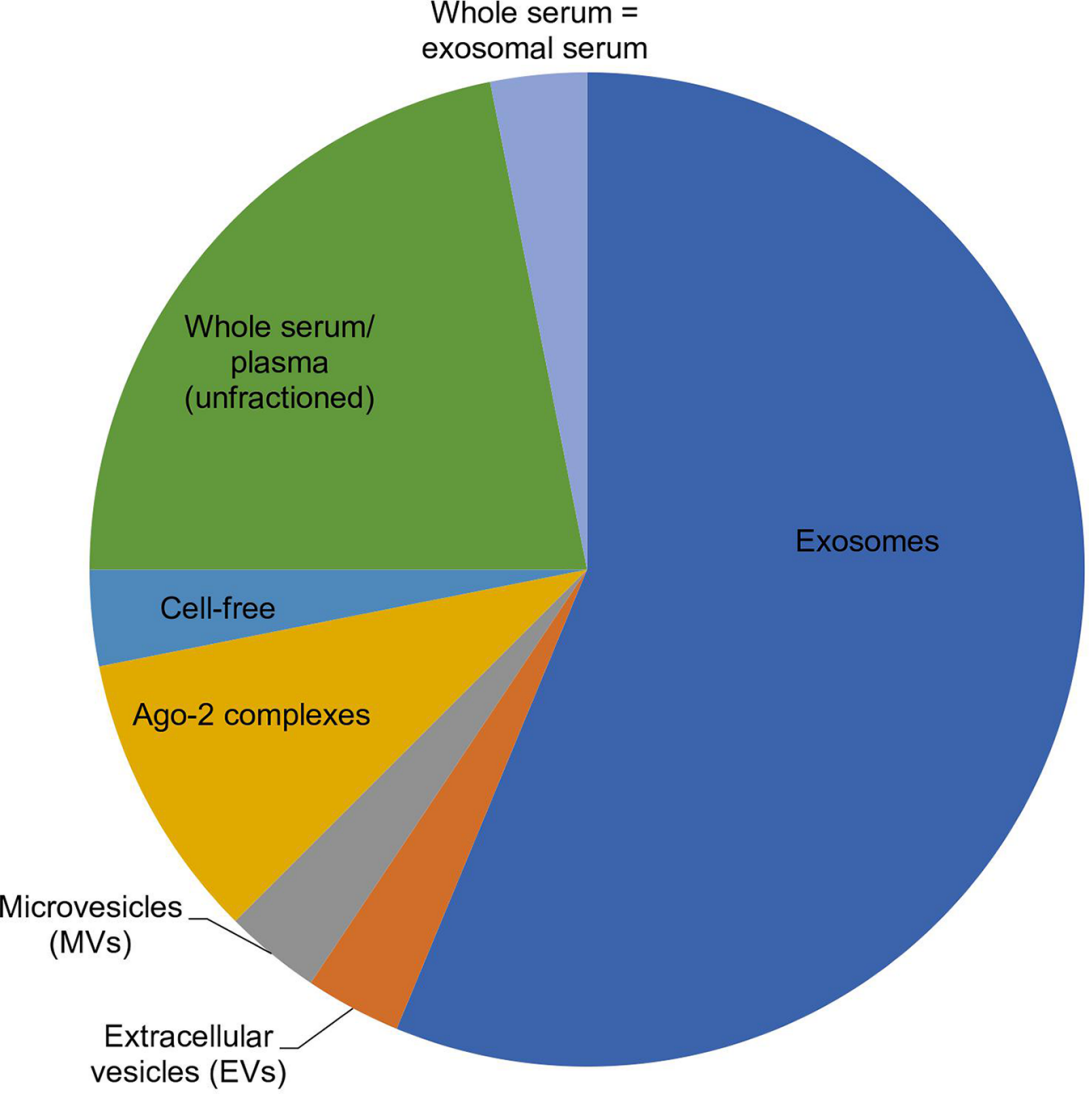
Pie chart showing the distribution of 32 selected articles in which a miRNA origin was preferred, according to their comparative study findings. Out of 32 articles, 18 chose exosomes, 1 chose EVs, 1 chose MVs, 3 chose Ago-2 complexes, 1 chose cell-free, 7 chose unfractioned, and 1 showed similar expression between whole serum versus exosomes, as the best origin for miRNAs used in biomarker studies.

Among the selected original articles, only six reported on non-exosomal circulating miRNAs: four studied microparticle (MP)-associated miRNAs, and two studied high-density lipoprotein (HDL)-associated miRNAs (**Figure 1**; **Table 1**). Meanwhile, a total of 31 of the original articles selected were exosomal miRNA studies: 15 used only plasma, 13 used only serum, 1 used both urine and plasma, 1 used plasma and serum, and 1 used only unstimulated whole saliva as the source of miRNAs for their study (**Figure 1**; **Table 2**). On the other hand, 32 of the original articles selected formed a group that were both exosomal and non-exosomal studies: 9 used only plasma, 14 used only serum, 1 used serum and saliva, 1 used vitreous humor and serum, 1 used whole blood and serum,2 used plasma and conditioned media, and 4 used serum and conditioned media as the source of circulating miRNAs (**Figure 1**; **Tables 3** and **4**).

**Table 1:** Summary of original articles involving non-exosomal circulating miRNAs as biomarkers.

Reference	Source of miRNAs	MP/HDL isolation method	miRNA isolation method	miRNA-related experiment	Highlighted miRNAs	Potential biomarker	Other information
Giannella et al., 2017	Endothelial microparticles (EMPs) - platelet-poor plasma	Centrifugation at 18,000 g	RNeasy MinElute cleanup kit^f^	RT-qPCR	miR126-3p	Vascular endothelial repair in different glucose tolerance	Downregulation in hyperglycemic condition resembling vascular protection of miR-126-3p is lost in early phases of glucose intolerance
Zhang et al., 2017	EMPs - plasma	Centrifugation at 13,000 g	NA	RT-qPCR	miR-92a	Early detection of acute myocardial infarction (AMI); differentiation from stable coronary artery disease (SCAD)	1. Upregulation pattern: AMI > SCAD > control 2. miRNA expression parallel with increase in CD31^+^/CD42b^−^ MPs 3. AUC of EMPs, miR-92a, and cTnI were 8.925, 8.883, and 9.123, respectively, suggesting combination usage for a more specific and sensitive biomarker
Jansen et al., 2016	MP-containing and cell-free plasma	Centrifugation at 13,000 g	TRIzol-based miR isolation	RT-qPCR	miR-126 and miR-26	SCAD	1. Downregulation of miRNAs in diabetic vs. non-diabetic patients 2. Downregulation leads to high risk of coronary artery disease (CAD) 3. MP plasma miRNAs were better than cell-free miRNAs in predicting cardiovascular events in SCAD patients
Niculescu et al., 2015	HDL - serum	Isopycnic density gradient UC	miRNeasy serum/ plasma kit^f^	1. Pathway-focused Human CVD miScript miRNA PCR array^f^ 2. RT-qPCR	miR-486 and miR-92	Vulnerable CAD	Upregulation in vulnerable CAD vs. stable CAD
	HDL_2_ - serum				miR-486		Upregulation in both unstable vagina (UA) and myocardial infarction (MI) patients; can differentiate between CAD and stable CAD
	HDL_3_ - serum				miR-92		
Diehl et al., 2012	MPs - platelet	Centrifugation at 16,000 g	Trizol	1. RT-qPCR 2. Next-generation sequencing^c^	miR-19, miR-21, miR-132, miR-133, miR-146, miR-155, miR-223, miR-377, miR-451, miR-641, miR-887, miR-1246, and miR-4284	CAD	miRNAs were associated with MP-containing plasma compared to MP-free plasma of CAD patients
Tabet et al., 2016	HDL - serum	PEG-6000 method	Qiazol miRNA-easy kits^f^	RT-qPCR	miR-223	Weight loss	Downregulation in HP patient vs. normal protein diet
					miR-16 and Mir-122		According to previous study: downregulation in gastric bypass bariatric surgery patients

^c^Illumina; ^f^Qiagen.

AMI, acute myocardial infarction; AUC, area under the curve; CAD, coronary artery disease; EMP, endothelial microparticle; HDL, high-density lipoprotein; HP, high protein; hsa, “homo sapiens” = human microRNA prefix; let, microRNA prefix that is conserved in many species; miRNA, microRNA; miR, mature form of microRNA, NA, not available; PEG, polyethylene glycol; RT-qPCR, real-time reverse transcription polymerase chain reaction; SCAD, stable coronary artery disease; UA, unstable vagina; UC, ultracentifugation.

**Table 2:** Summary of original articles involving exosomal miRNAs as biomarkers.

Reference	Source	Exosome isolation method	miRNA isolation method	miRNA-related experiment	Highlighted miRNAs	Potential biomarker	Other information
Fu et al., 2018	Serum	qEV size exclusion columns^a^	HiPure liquid RNA/ miRNA kit^b^	1. Small RNA sequencing^c^ 2. Stem-loop RT-qPCR	miR-17-5p and miR-92a-3p	Primary vs. metastatic colorectal cancer (CRC)	Upregulation of miRNAs is associated with pathologic stages and grades of CRC
Li et al., 2017	Plasma	ExoCap exosome isolation and enrichment kit^d^	NA	RT-qPCR	miR-96-5p and miR-149	Stage III CRC	
Yan et al., 2017	Serum	Total exosome isolation kit^e^	Trizol LS of miRNeasy mini kit^f^	1. Microarray^g^ 2. RT-qPCR	miR-638	CRC	Downregulation of miRNA associated with increase in liver metastasis and later tumor/nodes/metastasis (TNM) stage of CRC
					miR-548c-5p, miR-638, miR-5787, miR-8975, and miR-6869-5p		Involved in the process of glucose metabolism in CRC
Ostenfeld et al., 2016	Pooled serum; pooled plasma	Ultracentrifugation (UC)	miRNeasy mini kit^f^	miRCURY LNA™ universal RT microRNA PCR analysis	miR-16-5p, miR-23a-3p, miR-23b-3p, miR-27a-3p, miR-27b-3p, miR-30b-5p, miR-30c-5p, and miR-222-3p	CRC	miRNAs were tumor originated
Ogata-Kawata et al., 2014	750 µl serum	UC	Trizol-LS reagent + RNeasy mini spin columns^f^	1. Microarray^h^ 2. RT-qPCR	miR-23a	Early-stage CRC	95% sensitivity for stage 1
					miR-1246		90% sensitivity for stage 1
Wang et al., 2017	Plasma	ExoQuick^i^	miRNeasy micro kit^f^	RT-qPCR	miR-125a-3p	Colon cancer	1. Upregulation in patients with early-stage colon cancer 2. miRNA AUC = 68.55%; existing marker-CEA AUC = 83.6%; combination miR-125a-3p and carcinoembryonic antigen (CEA) AUC = 85.5% 3. miR-125a-3p = significant correlation with only nerve infiltration (P < 0.01), but CEA level correlated with tumor size, infiltration depth, and differentiation degree (P < 0.05, r = 0.3009-0.7270)
Teng et al., 2017	Serum	ExoRNeasy serum/ plasma midi kit^f^	miRNeasy mini kit^f^	qPCR	mir-193a	Tumor progression of colon cancer	Upregulation of miRNA shown in human colon cancer patients with more advanced disease
Kimura et al., 2018	Plasma	PEG system	Plasma/ Serum Circulating and Exosomal RNA Purification kit^j^	Real-time PCR	let-7i	Multiple sclerosis (MS)	Regulates MS pathogenesis by blocking the IGF1R/TGFBR1 pathway
Ebrahimkhani et al., 2017	1000 µl serum	RNase + SEC (Qev^a^)	Plasma/ Serum Circulating and Exosomal RNA Purification Mini kit^j^	Small RNA sequencing^c^	miR-15b-5p, miR-23a-3p, miR-30b,-5p, miR-223-3p, miR-342-3p, miR-374-5p, miR-432-5p miR-433-3p, and miR-485-3p	Relapsing-remitting MS vs. progressive MS	
Rani et al., 2017	Plasma	ExoEasy Maxi and exoRNeasy kit^f^	ExoRNeasy serum/ plasma maxi kit^f^	Small RNA sequencing	miR-10a-5p, miR-10b-5p, miR-23b-3p, miR-26b-5p, miR-30c-5p, miR-99a-5p, miR-125a-5p, miR-125b-5p, miR-140-3p, miR-186-5p, miR-378a-3p, miR-451a, and miR-342-3p	Cognitive decline in normal aging/presymptomic stage of disease	Monteral Cognitive Assessment (MoCA) scores were negatively correlated with 13 miRNAs, but the miRNA profile was different to previous reported in Alzheimer's disease
Ramachandran et al., 2017	100 µl plasma	ExoQuick^i^	SeraMir RNA isolation kit^i^	Ross score	miR-129-p	Heart failure (HF) in univentricular heart disease independent of ventricular morphology or stage of palliation	miRNA level is inversely related to the degree of clinical heart failure as assessed by Ross score
Melman et al., 2015	Plasma	UC	mirVana PARIS RNA isolation kit^e^	1. Megaplex miRNA primer pool^k^ 2. RT-qPCR	miR-30d	HF disease management	Upregulation of miRNA in coronary sinus (CS) patient with HF
Perge et al., 2017	200 µl plasma	Total exosome isolation (from plasma) kit^e^/UC	Total exosome RNA and protein isolation kit^e/^MinElute cleanup it^f^	1. miRNA profiling: Megaplex pools 2. RT-qPCR	miR-483-5p	Adrenocortical cancer (ACC) vs. adrenocortical adenomas (ACA) preoperative diagnosis of ACC	Upregulation of miRNA in ACC compared to ACA, with AUC 0.965, sensitivity of 87.5%, and specificity 94.45
Murray et al., 2017	800 µl serum	PEG	Trizol LS^l^	RT-qPCR	miR-122 and miR-200a	Liver disease	Upregulation of miRNAs in anti-retroviral therapies (ART)-treated, HIV-1 infected individuals prior to the development of fatal liver disease
Momen−Heravi et al., 2015	150 μl plasma	ExoQuick^i^	QIAzol Lysis + Direct-zol™ RNA MiniPrep isolation kit^m^	1. Microarray^n^ 2. RT-qPCR	miR-30a and miR-192	Liver disease	1. Upregulation of miRNAs in alcoholic patients 2. miRNA-192 has AUC = 0.95; p < 0.001
Sugimachi et al., 2015	Serum: 1500 µl (microarray) and 300 µl for RT–qPCR	Filtration through 0.22-mm filter + UC	miRNeasy mini kit^f^	1. Microarray (3D-gene human miRNA oligo chips) 2. RT-qPCR	miR-718	Recurrence and therapeutic targets of hepatocellular carcinoma (HCC)	Downregulation in patients with tumor recurrence after liver transplant (LT)
Liu et al., 2017a	300 µl plasma	ExoQuick^i^	NA	1. miRNA profiling ° 2. RT-qPCR	miR-10b-5p, miR-21-5p, and miR-23b-3p	Non-small-cell lung cancer (NSCLC)	Upregulation of miRNAs were independently associated with poor survival in NSCLC patients
Cazzoli et al., 2013	500 µl plasma	ExoQuick^i^	Rneasy Lysis Buffer RLT^f^ + Trizol^l^	qRT-PCR	miR-139-5p, miR-200b-5p, miR-378a, and miR-379	Screening test: nodule (lung adeno-carcinomas +carcinoma) vs. non-nodule (healthy former smokers) lung adeno-carcinomas + carcinoma	97.5% sensitivity, 72% specificity, and AUC ROC of 90.8%
					miR-30a-3p, miR-100, miR-151a-5p, miR-154-3p, and miR-200b-5p	Diagnostic test: Only nodule population Lung adenocarcinoma vs. granuloma	96% sensitivity, 60% specificity, and AUC ROC of 76%
Kobayashi et al., 2017	1000 µl unsti-mulated whole saliva	Total exosome isolation reagent^e^	Total exosome RNA and protein isolation kits^e^	1. PCR array analyses and data analysis: neuropathic and inflammatory miScript miRNAs PCR array^p^ 2. RT-qPCR	miR203a-3p	Low oral health-related quality of life (OHRQoL) group vs. high-OHRQoL	Upregulation of miRNA in low-OHRQoL
Kangas et al., 2017	450µl serum	ExoQuick^i^	Trisure reagent	1. qPCR 2. small RNA sequencing^c^ 3. RT-qPCR	miR-27-3p, miR-28-3p, miR-30a-5p, miR-106-5p, miR-126-5p, and miR-148a-3p	Metabolic and inflammatory status as a result of hormonal changes at menopause	miRNA levels significantly associated with serum 17β-estradiol
Domanska-Senderowska et al., 2017	200 µl serum	Total exosome isolation reagent (from serum)^e^	Total exosome RNA and protein isolation kit^e^	RT-qPCR	miR-27b and miR-29	Cardio-respiratory fitness	Upregulation of miRNAs
Chen et al., 2017	500 µl serum	ExoQuick^i^	miRNeasy mini kit^f^	qPCR	miR-223	Acute ischemic stroke occurrence, stroke severity, and short-term outcomes	Upregulation of miRNAs
Yuan et al., 2016	plasma	ExoQuick^i^ + RNAse A	miRNeasy micro kit^f^	Small RNA sequencing^c^	miR-125a and miR-1343-3p	Colon cancer; prostate cancer including castration-resistant and hormone-sensitive; pancreatic cancer	1. Down-regulation with all cancer types tested with <0.05 false rate 2. AUC 0.68 to 0.92, depends on cancer type and stage
Meng et al., 2016	600 μl serum	Total exosome isolation reagent for serum^e^	mirVana PARIS kit^e^	RT-qPCR	miR-373	More aggressive breast carcinomas	Association of levels with triple negative
Hannafon et al., 2016	Plasma	ExoQuick^i^	TRIzol	1. Next-generation sequencing^c^ 2. RT-qPCR	miR-21 and miR-1246	Breast cancer (accompany another diagnostic tool)	AUC 0.73 (95% CI 0.53, 0.92; P = 0.022)
Khalyfa et al., 2016	Plasma	Total exosome isolation reagent^e^	miRNeasy mini kit^f^	Microarrays^h^	hsa-miR-483-3p	Intermittent hypoxia (IH) exposure	Exosomal role as cell-to-cell communicator
Alhasan et al., 2016	1000 µl serum	ExoQuick^i^	mirVana PARIS miRNA isolation kit^e^	1. miRNA profiling (scano-miR platform) 2. RT-qPCR	Prostate cancer (Pca) miRNAs: miR-106a, miR-135a*, miR-200c, miR-433, and miR-605	Very high-risk (VHR) Pcas patients vs. low-risk patients and healthy subjects VHR aggressive Pca	Differentiate population with at least 89% accuracy
Huang et al., 2015	250 µl plasma	ExoQuick^i^+ RNase A	miRNeasy micro kit^f^	RT-qPCR	miR-375 and miR-1290	Castration-resistant prostate cancer (CRPC)	Upregulation of miRNAs significantly associated with overall survival
Ramezani et al., 2015	500 µL plasma	Exosome RNA isolation kit^j^	NA	1. Microarrays^g^ 2. RT-qPCR	miR-30b, miR-30c, miR-34b, miR-34c, and miR-342	Minimal change disease (MCD) patients vs. focal glomerulo-sclerosis (FSGS) patients and control subjects	Upregulation of miRNAs
	1000 µL urine				miR-1225-5p		
					miR-1915 and miR-663	FSGS vs. MCD and control subjects	Downregulation of miRNAs
					miR-155		Upregulation of miRNAs
Hubert et al., 2015	250µl plasma	ExoQuick^i^	TRIzol LS^l^	RT-qPCR	miR-155 and miR-223	HIV-1 pathogenesis, disease progression, association with inflammatory state, and efficacy of anti-retrovirus therapy (ART) treatments	miRNA expression strongly correlated with exosome abundance and size in ART-naïve patients
Ye et al., 2014	250 µL serum	UC	TRIzol^q^ + miRN-easy mini kit^f^	microarray°	hsa-miR-20a-5p, hsa-miR-24-3p, hsa-miR-891a, hsa-miR-106a-5p, and hsa-miR1908	T-cell function in nasopharyngeal carcinoma (NPC)	Upregulation of miRNAs in NPC patients leads to downregulation of the MARK1 signaling pathway and alters cell proliferation and differentiation.

^a^Izon Science Ltd.; ^b^Magen, China; ^c^Illumina; ^d^JSR Micro Materials Innovations; ^e^Ambion; ^f^Qiagen; ^g^Affymetrix; ^h^Agilent; ^i^System Biosciences; ^j^Norgen; ^k^Applied Biosystems; ^l^Life Technologies; ^m^Zymo Research Corp; ^n^Firefly BioWorks; °Exiqon; ^p^SA biosciences; ^q^Invitrogen.

ACA, adrenocortical adenomas; ACC, adrenocortical cancer; ART, anti-retroviral therapies; AUC, area under the curve; CRC, colorectal cancer; CRPC, castration-resistant prostate cancer; CS, coronary sinus; FSGS, focal segmental glomerulosclerosis; HCC, hepatocellular carcinoma; HF, heart failure; hsa, “homo sapiens” = human microRNA prefix; IH, intermittent hypoxia; let, microRNA prefix that is conserved in many species; LT, liver transplant; OHRQoL, oral health-related quality of life; MCD, minimal change disease; miRNA, microRNA; miR, mature form of microRNA, MoCA, Montreal Cognitive Assessment; MS, multiple sclerosis; NA, not available; NPC, nasopharyngeal carcinoma; NSCLC, non-small cell lung cancer; PEG, polyethylene glycol; PCa, prostate cancer; qPCR, quantitative polymerase chain reaction; ROC, receiver operating curve; RT-qPCR, real-time reverse transcription polymerase chain reaction; UC, ultracentifugation; VHR, very high risk.

**Table 3:** Summary of articles with comparable performance between exosomal, non-exosomal circulating miRNAs, and unfractioned miRNA samples as potential biomarkers.

Reference	Sources of miRNAs	Exosomal & non-exosomal isolation method	miRNA isolation method	miRNA-related experiment	Highlighted miRNAs	Better miRNA source according to findings	Potential biomarker
Van Eijndhoven et al., 2016	Tumor cells vs. whole plasma vs. EVs plasma	Size exclusion chromate-graphy (SEC)	Trizol-LS^l^	1. Small RNA sequencing^c^ 2. Multiplex stem-loop RT-PCR	miR-24-3p, miR-127-3p, miR-21-5p, miR-155-5p, and let-7a-5p	EV plasma	Hodgkin's disease
Beg et al., 2017	(200 µl): whole plasma vs. exosomal plasma	Exosome RNA isolation kit	mirVana miRNA isolation kit^e^	RT-qPCR	miR-146a	Exosomal plasma	Heart failure (HF)
Crossland et al., 2017	(250 µl): whole serum vs. exosomal serum	Total exosome isolation reagent^l^	Total RNA Isolation kit^j^	RT-qPCR	miR-423, miR-199 and miR-93*	Exosomal serum	Acute graft-versus-host disease
de Gonzalo-Calvo et al., 2017	Whole serum validated with cultured cells vs. exosomal conditioned media	NA	mirVana kit^e^	RT-qPCR	miR-1 and miR-133a	Whole serum	Subclinical diabetic cardio-myopathy
Endzeliņš et al., 2017	(400 µl): whole plasma vs. EV plasma	SEC	Modified miRN-easy Micro Kit^f^ 5 volumes of QIAzol Lysis	RT-qPCR	miR-375	Whole plasma	Prostate cancer (Pca) vs. benign prostatic hyperplasia
					miR-200c-3p and miR-21-5p	EV plasma	
Li et al., 2016	(400 ml): whole serum vs. exosomal serum	ExoQuick^i^	miRNeasy serum/ plasma kit^f^	RT-qPCR	miR-141	Exosomal serum	Pca
Fujiwara et al., 2017	800 µl whole serum validated using cell culture and exosomal conditioned media	NA	miRNeasy RNA isolation kits^f^	1. Microarray^h^ 2. RT-qPCR	miR-17-5p and miR-25-3p	Whole serum: based on validated cultured studies	Osteosarcoma
Huang et al.,2017	Tissues, 200 µl arterial whole serum, 200 µl exosomal serum	ExoQuick^i^	mirVana PARIS kit^e^	RT-qPCR	MiR-20b-5p, miR-28-3p, and miR-192-5p	Similar performance; need thorough study using other vesicles and complexes to make comparisons	esophageal squamous cell carcinoma (ESCC)
Khare et al., 2017	425 µl whole plasma vs. exosomal plasma	UC	RNeasy MinElute columns^f^	Small RNA sequencing^c^	miR-12, miR-23a, miR-25, miR-26b, miR-30a, miR-30d, miR-93, miR-140*, miR-122, miR-144, miR-182, and miR-186	Whole plasma	Relapse lymphoma
Liu et al., 2017b	Whole serum vs. exosomal serum vs. cell-free serum	ExoQuick^i^	mirVana™ miRNA isolation kit^e^	RT-qPCR	miR-125b	Exosomal serum	Recurrence and survival of hepatocellular carcinoma (HCC)
Fornari et al., 2015	(400 μl): exosomal serum vs. non-exosomal serum	NA	Trizol reagent^l^	1. Microarray 2. RT-qPCR	miR-519d, miR-21, and miR-221	Exosomal serum	HCC
Sohn et al., 2015	(500 μl): whole serum vs. exosomal serum	ExoQuick^i^	miRNeasy serum/plasma kit^f^	RT-qPCR	miR-18a, miR-101, miR-106b, miR-122, miR-195, miR-221, miR-222, and miR-224	Exosomal serum	HCC
Moen et al., 2017	Cells vs. 200 µl exosomal serum	NA	miRNeasy serum plasma isolation kit^f^	RT-qPCR	miR-223, miR-760, and miR-145	Exosomal miRNAs showed to be released from cells asso-ciated with upregulation of inflammation	Chronic lumbar radicular pain
Rabinowits et al., 2017	Tissues vs. exosomal plasma vs. cell-free plasma	ExoQuick^i^	TRIzol reagent	TaqMan-based miRNA profiling: TaqMan array human miRNA platform	hsa-miR-19a, hsa-miR-512-3p, hsa-miR-27b, hsa-miR-20a, hsa-miR-28-3p, hsa-miR-200c, hsa-miR-151-3p, hsa-miR-223, hsa-miR-20b, hsa-miR-22, hsa-miR-516-3p, hsa-miR-370, hsa-miR139-5p, hsa-miR-145-3p, hsa-let-7e, and hsa-miR-30c	Exosomal plasma	Squamous cell carcinoma
Reithmair et al., 2017	3 000 µl: whole blood vs. whole serum vs. exosomal serum	miRCURY exosome isolation kit°	miRCURY RNA isolation kit-biofluids°	Next-generation sequencing^c^	miR-193a-5p and miR-125-5p	Exosomal serum	Septic shock
Uotani et al., 2017	(200 µl): exosomal serum vs. Ago2 complexes	SEC	MiRNeasy mini kits^f^	1. Microarray^h^ 2. RT-qPCR	miR-92B-3p	Exosomal serum	Synovial sarcoma (SS)
Yokoi et al., 2017	200 µL (RT-qPCR)/ 600 µL (NGS): whole serum validated in exosomal conditioned media vs. cultured cells	NA	QIAzol and the miRNeasy mini kit^f^	1. Small RNA sequencing^c^ 2. RT-qPCR	miR-142-3p, miR-26a-5p, miR-374a-5p, miR-766-3p, miR-200a-3p, miR-328-3p, miR-130b-3p, and let-7d-5p	Circulating miRNAs in whole serum were believed to be exosomal-originated	Ovarian cancer
Dinh et al., 2016	(200 μl): whole plasma validated with cultured cells vs. exosomal conditioned media	NA	Exiqon miRCURY biofluids total RNA isolation kits	1. miRNA profiling° 2. RT-qPCR	miR-29a-3p, miR-150-5p	Whole plasma	Thoracic radiation therapy for non-small cell lung cancer
Emanueli et al., 2016	(100 μl): whole plasma vs. exosomal plasma	Exo-spin mini-columns^r^	miRNeasy kit^f^	microRNA analyses	Cardiac expressed miRs: miR-1, miR-24, miR-133a/b, miR-208a/b, and miR-210 Non-vascular miR-122	Exosomal plasma	Myocardial damage
Uratani et al., 2016	(250 μl): whole serum vs. exosomal serum	ExoQuick^i^	miRNeasy kit^f^	RT-qPCR	miR-21, miR-29a, and miR-92a	Whole serum	Large adenomatous polyps
Pirola et al., 2015	(200 µl): whole serum vs. Ago2 complexes serum vs. cell-free and Ago2-free serum	NA	miRNeasy serum/plasma kit^f^	1. Global profiling 2. In-situ hybridization (ISH) 3. RT-PCR 4. Cell culture and transfection	miR-122	Ago2-free serum	Non-alcoholic fatty liver disease (NAFLD)
Ragusa et al., 2015	Vitreous humor (VH) vs. exosomal VH vs. (400 µL): whole serum vs. exosomal serum	UC	miRNeasy mini kit^f^	miRNA profiling (TaqMan low density array)	miR146a	Similar expression	Uveal melanoma (UM)
Eichelser et al., 2014	(400 μL): whole serum vs. exosomal serum vs. cell-free serum	ExoQuick^i^	mirVana PARIS kit^e^	1. qRT-PCR 2. Luciferase assay^s^	miR-101 and miR-373	Exosomal serum	Aggressive breast carcinomas
Jansen et al., 2014	(250 µl): MVs plasma vs. cell-free plasma	Centrifugation at 13,000 g	TRIzol LS^l^	RT-qPCR	miR-126 and miR-199a	MV plasma	Cardiovascular events in stable coronary artery patients
Kubiczkova et al., 2014	(250 µl): whole serum vs. exosomal serum vs. cell-free serum	ExoQuick^i^	modified miRNeasy kit^f^	1. Mega-plex profiling 2.qPCR	miR-744, miR-130a, miR-34, let-7d, and let-7e	Whole serum	Multiple myeloma and monoclonal gammapathy
Chugh et al., 2013	(250 µl): exosomal plasma vs. Ago2 complexes	ExoQuick^i^	TRI reagent	RT-qPCR	miR-17-92	Exosomal plasma	Kaposi's sarcoma (KS)
de Candia et al., 2013	(400 µl): whole serum vs. exosomal serum	UC/ ExoMir filters	mirVana miRNA isolation kit^e^	RT-qPCR: stem-loop RT-qPCR and Exiqon locked nucleic acid (LNA)-based RT-qPCR	miR-150	Exosomal serum	Immunization

^c^Illumina; ^e^Ambion; ^f^Qiagen; ^h^Agilent; ^i^System Biosciences; ^l^Life Technologies; °Exiqon; ^r^Cell guidance system; ^s^Promega.

ESCC, esophageal squamous cell carcinoma; EV, extracellular vesicle; HCC, hepatocellular carcinoma; hsa, “homo sapiens” = human microRNA prefix; KS, Kaposi's sarcoma; LNA, locked nucleic acid; let, microRNA prefix that is conserved in many species; miRNA, microRNA; miR, mature form of microRNA, NA, not available; NAFLD, non-alcoholic fatty-lipid disease; PCa, prostate cancer; RT-qPCR, real-time reverse transcription polymerase chain reaction; SEC, size-exclusion chromatography; SS, Synovial sarcoma; UC, ultracentifugation; UM, uveal meloma; VH, vitreous humor.

**Table 4:** Summary of articles using different sources of miRNAs in discussing subjects other than potential biomarkers of a particular disease.

Reference	Sources of miRNAs	Exosomal & non-exosomal isolation method	miRNA isolation method	miRNA-related experiment	Highlighted miRNAs	Better miRNA source according to findings	Potential biomarker
Koberle et al., 2013	(200 µL): exosomal serum vs. cell-free serum	UC	miRNeasy mini Kit^f^	RT-qPCR	miR-1, miR-16, miR-21, miR-122 and miR-142-3p	Exosomal serum	1. Exosomal miRNAs are more stable than cell-free miRNAs 2. RNase inhibitor can be used to differentiate between exosomal and cell-free miRNAs faster and more easily than UC
Gallo et al., 2012	Whole serum vs. exosomal serum vs. cell-free serum	UC	Trizol^q^	RT-qPCR	let-7a; miR-92a, miR-142-3p, miR-101, miR-16, miR-107, miR-122, miR-547, and miR-768	Exosomal serum	Majority of miRNAs in serum and saliva are concentrated in exosomes
	Whole saliva vs. exosomal saliva vs. cell-free saliva				miR-22, miR-202, miR-203, and miR-1273d	Exosomal saliva	
Li et al., 2012	Exosomal plasma vs. Ago2 complexes	Differential centrifugation and UC	Trizol^q^/ miRNeasy mini kit^f^	RT-qPCR	miR-16	Ago2 complexes	1. Vesicle structures such as exosomes provide general protection to vesicle-encapsulated miRNAs. 2. Ago2 complexes selectively associated with miRNAs in vesicles under functional status and protect the vesicle miRNAs from degradation by RNases or proteases.
	Exosomal conditioned media vs. Ago2 complexes conditioned media						
Arroyo et al., 2011	Exosomal plasma vs. Ago2 complexes plasma	UC/SEC	miRNeasy kit^f^	RT-qPCR	miR-16, miR-92a, and let-7a	Ago2 complexes plasma	Two populations of circulating miRNAs exist in plasma: Ago2 complexed and vesicles (exosomes)-bound; suggested that circulating Ago2 complexes are a mechanism responsible for the stability of plasma miRNAs
Turchinovich et al., 2011	(400 µl): exosomal plasma vs. Ago2 complexes plasma	UC	Tri-Reagent LS^t^ + miRNeasy kit^f^	qRT-PCR	hsa-miR-16, has-miR-21, and has-miR-24	Ago2 complexes plasma	Majority of nuclease-resistance extracellular miRNA in plasma and cell culture media is floating outside exosomes and is bound to Ago2 protein
	Exosomal conditioned media vs. Ago2 complexes conditioned media					Ago2 complexes conditioned media	

^f^Qiagen; ^q^Invitrogen; ^t^Sigma.

Ago, Argonoute; hsa, “homo sapiens” = human microRNA prefix; let, microRNA prefix that is conserved in many species; miRNA, microRNA; miR, mature form of microRNA, NA, not available; RT-qPCR, real-time reverse transcription polymerase chain reaction; SEC, size-exclusion chromatography; UC, ultracentifugation.

## Biomarker

A biomarker is defined as an objectively measured and evaluated indicator of normal biological processes, pathogenic processes, or pharmacological responses to therapeutic intervention. A biomarker can thus provide impartial information regarding the current physiological state of living organisms (Gill et al., 2017). Biomarkers exist in the form of antibodies (Dardari et al., 2000: IgG-ZEBRA as a biomarker in young patients with nasopharyngeal carcinoma), microbes (Shah et al., 2018: the fecal microbiome as a biomarker of colorectal cancer), DNA (Teo et al., 2019: sequencing of circulating cell-free DNA [cfDNA] from human blood plasma as a noninvasive methodology to study age-associated changes in the epigenome *in vivo*), RNA/miRNA (as tabulated in this review), lipids (Seow et al., 2019: the lipid fraction of the solid non-enhancing region showed potential as a novel prognostic biomarker of survival outcome in glioma), metabolites [Lou et al., 2019: a urinary metabolite of polycyclic aromatic hydrocarbons, 8-hydroxy-2′-deoxyguanosine (8-OHdG), can be a biomarker of oxidative stress in pregnant women], and/or protein [Xu et al., 2019: serum protein, the insulin-like growth factor-binding protein 1 (IGFBP-1), as a biomarker for the early detection of gastrointestinal cancer]. Alteration in biomarker concentration, structure, function, or action can be associated with the onset, progression, or even regression of a particular disorder as a result of how the body responds to it (Gill et al., 2017).

A biomarker needs to pass several tests before it can be successfully used clinically. These tests are (1) preclinical testing, performed using patient samples and confirmed at the *in vitro* and *in vivo* levels, (2) feasibility analysis, which involves the usage of small patient subpopulations to demonstrate the ability to discriminate diseased from healthy subjects, (3) the validation process, which is used to confirm that the biomarker has been assayed properly, and (4) statistical analysis, which is carried out to evaluate the discriminatory accuracy of the biomarker in a large patient population (Gill et al., 2017).

## MicroRNA (miRNA)

### Origin

miRNAs can originate from intergenic or intragenic (both exonic and intronic) genomic regions, transcribed into long primary transcripts called pri-miR, which are folded back and form double-stranded hairpin structures. The pri-miR will be subjected to a sequential process to produce miRNAs. First is the production of precursor molecules called pre-miR (80-120 nucleotides) in the nucleus by type III endonuclease DROSHA. These pre-miRs are then exported to the cytoplasm, mediated by EXPORTINS, and are later processed by another type of endonuclease, DICER, into short “active” molecules called miRNAs (Redis et al., 2012). These mature miRNAs will then be integrated into the RNA-induced silencing complex (RISC) to exhibit their role as gene expression regulators (Mo et al., 2012; O'Brien et al., 2018).

### Source of Circulating miRNAs

Circulating miRNAs, which are found in body fluids such as in saliva, serum, plasma, and milk, can be secreted or produced as a result of various events, such as (1) passive leakage from cells that suffer injury, chronic inflammation, apoptosis, or necrosis, or perhaps from cells with a short half-life like platelets, (2) active secretion *via* cell-derived membrane vesicles such as microparticles (MPs), exosomes, shedding vesicles, and apoptotic bodies, and (3) active secretion by a protein-miRNA complex, whereby miRNAs build an association with either or both of lipoproteins (e.g., high-density lipoprotein: HDL) and Argonaute protein (e.g., Ago2) (Redis et al., 2012).

### Factors Contributing to the Stability of Circulating miRNAs and Its Benefits to Biomarker Development

Circulating miRNAs are unusually stable due to (1) their packaging in vesicles, such as in exosomes (Chugh et al., 2013) or apoptotic bodies, (2) their RNA folding and size, and/or (3) their presence in Ago-containing ribonucleic acid: protein (RNP) complexes (Chugh et al., 2013), with nucleophosmin (Kubiczkova et al., 2014), or with high-density lipoproteins (HDL) (Köberle et al., 2013; Fornari et al., 2015). The stability of miRNAs results from their resistance toward the action of ribonucleases (Jansen et al., 2014; Dinh et al., 2016; Beg et al., 2017; Liu et al., 2017a; Yokoi et al., 2017; de Candia et al., 2013; Li et al., 2012). This stability means that they are long-lived in biological fluids (Ragusa et al., 2015; Emanueli et al., 2016) and are therefore proposed as attractive diagnostic (Pirola et al., 2015) and prognostic biomarkers (Fornari et al., 2015). Their easy accessibility through the collection of body fluids such as blood derivatives, saliva, or urine also points toward their being an ideal source for miRNA biomarkers (Gallo et al., 2012). Several experimental studies have also reported resistance of endogenous circulating miRNAs toward severe stressing conditions, such as high temperatures, repeated freeze-thaw cycles (Fornari et al., 2015), very low or high pH levels (Duttagupta et al., 2011), boiling, and extended storage time (Uratani et al., 2016; Domańska-Senderowska et al., 2017; Fu et al., 2018), which are positive traits for circulating miRNAs as potential biomarkers.

Non-invasive or minimally invasive collection of circulating miRNAs (Duttagupta et al., 2011; Pirola et al., 2015; Ragusa et al., 2015; Dinh et al., 2016; Uratani et al., 2016; Perge et al., 2017) enables (1) the prevention of repetitive invasive procedures such as tissue biopsy (e.g., liver biopsy in detecting hepatocellular carcinoma (HCC) (Sohn et al., 2015), dangerous kidney biopsy in distinguishing minimal change disease (MCD) from focal segmental glomerulosclerosis (FSGS) (Ramezani et al., 2015), or bone marrow procedures (Kubiczkova et al., 2014), (2) the avoidance of possible difficulties in obtaining real samples from cirrhotic patients with liver nodules of uncertain malignancy that are identified *via* imaging techniques (Fornari et al., 2015), and (3) the avoidance of sampling limitations such lesions being too small to be biopsied and studied using conventional methods (Ramezani et al., 2015).

The availability and easy accessibility of circulating miRNAs enables them to be substitutes for existing tedious and expensive procedures that require a high degree of expertise such as X-ray, computed tomography (CT), positron emission tomography (PET)-CT, magnetic resonance imaging (MRI) (Fujiwara et al., 2017), sigmoidoscopy, colonoscopy (Uratani et al., 2016), ophthalmoscopy, ultrasonography, fundus fluorescein angiography, and indocyanine green angiography (Ragusa et al., 2015). In addition to this, they may fill the gaps where useful biomarkers are yet to be found, with higher specificity and sensitivity (if possible). In cases like FSGS and MCD, miRNA expression has been reported to be able to differentiate diseases that seem similar at first glance or are related to each other yet need different treatment for recovery, to avoid resistance buildup, and to avoid progression into end-stage disease or, at worst, mortality. miRNAs are also able to act as (1) a real-time monitor of drug response, (2) an early detector of recurrence or metastasis, and (3) a substitute for a known serum-based tumor marker of osteosarcoma (alkaline phosphatase [ALP]) that sometimes leads to false-positive results (since ALP is generally elevated in children and affected by organ damage) in detecting and monitoring the tumor burden of osteosarcoma (Fujiwara et al., 2017). Circulating miRNAs are also reported to be better markers than existing markers, for example, (1) cancer antigen-125 (CA-125) measurement and ultrasonography in detecting ovarian cancer (OvCa) (Yokoi et al., 2017) or (2) squamous cell cancer antigen (SCC) and carcinoembryonic antigen (CEA) (Huang et al., 2017).

### Limitations in miRNA Biomarker Studies

Nevertheless, although studies on the development of miRNAs as biomarkers are blooming, several limitations should be addressed. One of the main limitations is poor consensus between different studies on a particular disease. This may be due to a lack of standardization in the choice of control population, source of circulating miRNAs (serum/plasma/urine/saliva/etc), isolation protocol (Teng et al., 2017; Wang et al., 2017), choice of internal and exogenous control (Fornari et al., 2015; Ostenfeld et al., 2016; Yuan et al., 2016; Khare et al., 2017; Rabinowits et al., 2017), analytical approach (e.g., using microarray versus RT-qPCR), or sample size (Niculescu et al., 2015). The source of miRNAs being the cause of variation in results can be seen in a study reported by Huang et al. (2017; tissues versus circulating miRNAs) and a study by Reithmair et al. (2017; cellular versus vesicle or cell-free miRNAs). The second limitation is lacking a large sample size and validation of results in different independent cohorts (Gallo et al., 2012; Momen−Heravi et al., 2015; Dinh et al., 2016; Jansen et al., 2016; Beg et al., 2017; Huang et al., 2017; Khare et al., 2017; Liu et al., 2017a; Rabinowits et al., 2017; Yokoi et al., 2017). The sample size in biomarker studies is usually only suitable for a pilot study. The third limitation is a lack of detailed study of a particular disease. Limitations in financing, facilities, and sample sources can lead to an inability to perform all related experiments on a particular disease in order to reach a conclusive result. Hence, there is still a huge amount of room for uncertainty over whether the published results (Jansen et al., 2016) show the reality of the disease condition. The fourth limitation is the effect of external factors such as age, gender, the matched control, and other external factors (tobacco, alcohol, etc.); these are usually neglected, but they may contribute to variation in results (Rabinowits et al., 2017). Rani (2017) reported such a case, where hsa-let-7e-5p and hsa-miR-103a-3p expression were decreased while the expression of other miRNAs was elevated in either the blood or plasma of Alzheimer's patients. This situation emphasizes the importance of considering age when investigating biomarkers for a disease (Rani et al., 2017). The fifth limitation is an inability to specifically pinpoint the origin of the miRNAs. Most of the studies considering circulating miRNAs as biomarkers focused on using blood as the miRNA source. However, human blood contains a variety of cell types, hence making it challenging to identify the cell origin of the particular miRNA focused on in a study (Endzeliņš et al., 2017; Murray et al., 2017).

## Extracellular Vesicles (EVs)

Extracellular vesicles (EVs), which are produced by cells, can be divided into three main types according to their size and biogenesis: exosomes, microvesicles (MVs), and apoptotic bodies (refer to **Table 5** for distinctions between exosomes and other EVs). Previous studies have shown that EVs can be secreted by a variety of cell types and can be isolated from different sources such as blood, urine, breast milk, and saliva. EVs contain different types of molecules, such as DNA, RNA, miRNA, lipids, and proteins. EV membranes are known to protect their contents, for example, from degradation activities by nucleases and proteases and also from micro-environment changes (such as fluctuations in pH and osmolarity). EV contents can be transported from the origin parental cells to a specific recipient cell, either *via* horizontal transfer or *via* distant tissues. The easy accessibility of EVs for isolation, their stability, and the ability of their contents to be transported from cell to cell make EVs attractive sources for diagnostic and prognostic biomarkers (Kim et al., 2017).

**Table 5:** Distinctions between exosomes, microvesicles (MVs)/microparticles (MPs), and apoptotic bodies.

Extracellular Vesicles (EVs)			
Feature	Exosomes	Microvesicles (MVs)/Microparticles (MPs)	Apoptotic bodies
**Visual**	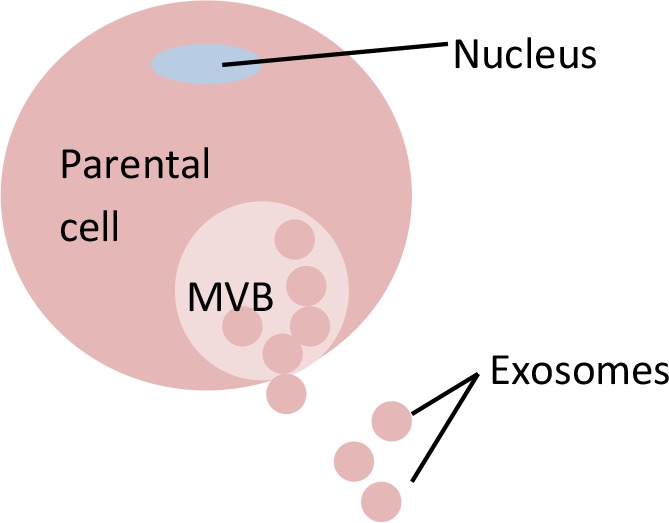	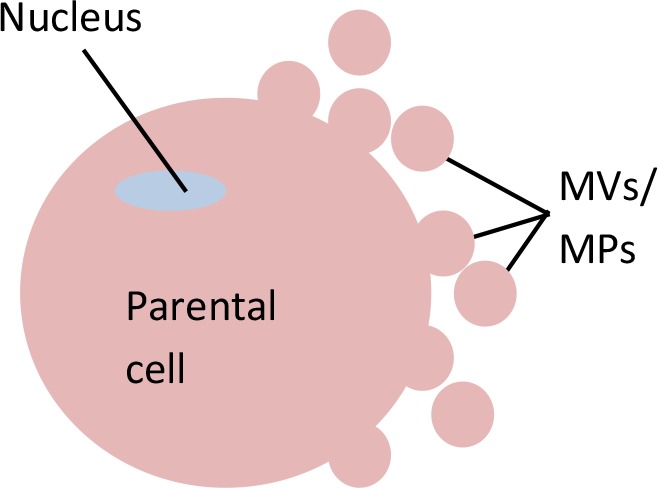	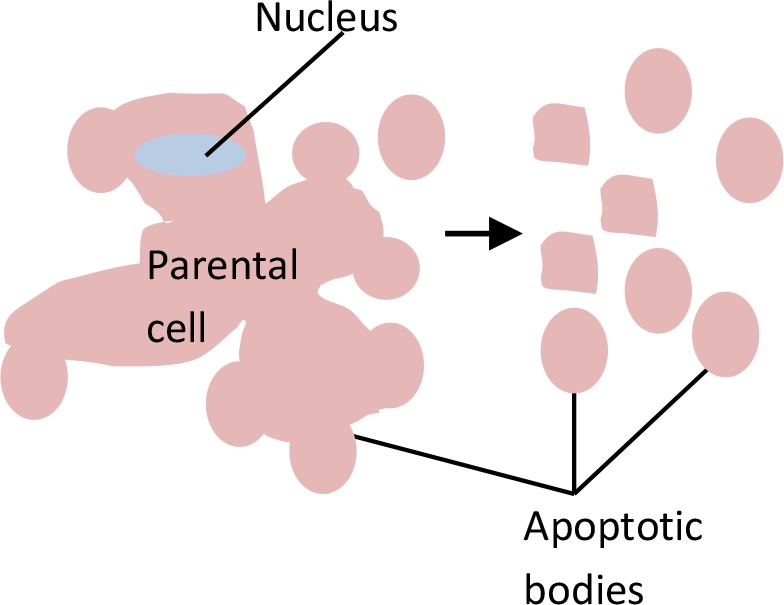
**Size (µm)**	0.03 - 0.1	0.1 - 1.0	1.0 - 5.0
**Markers**	CD9, CD63, CD81, Alix, TSG101	Annexin V, integrin, selectin, flotillin-2, CD40 ligand	Annexin V, DNA, histones
**Biogenesis**	MVB fusion with parental cell plasma membrane	Parental cell membrane blebbing	Apoptosis of parental cell
**Mechanism of release**	Exocytosis of MVBs	Budding of parental plasma membrane	Parental cell shrinkage, followed by blebbing of plasma membrane
**Detection method**	FACS with CD68 capture, electron microscopy, Western blot	FACS and electron microscopy	FACS and electron microscopy
**Standard isolation method**	Ultracentrifugation (≥100,000 g), immunoprecipitation (ExoQuick), Size exclusion chromatography (SEC)	Centrifugation 10,000-20,000 g	No known standard method
**References**	Mathivanan et al., 2012; Kim et al., 2017; Uotani et al., 2017; Chen et al., 2018	Jayachandran et al., 2012; Kim et al., 2017; Gianella et al., 2017; Chen et al., 2018	Kim et al., 2017; Chen et al., 2018; Hristov et. al., 2004

**Figure 2:**
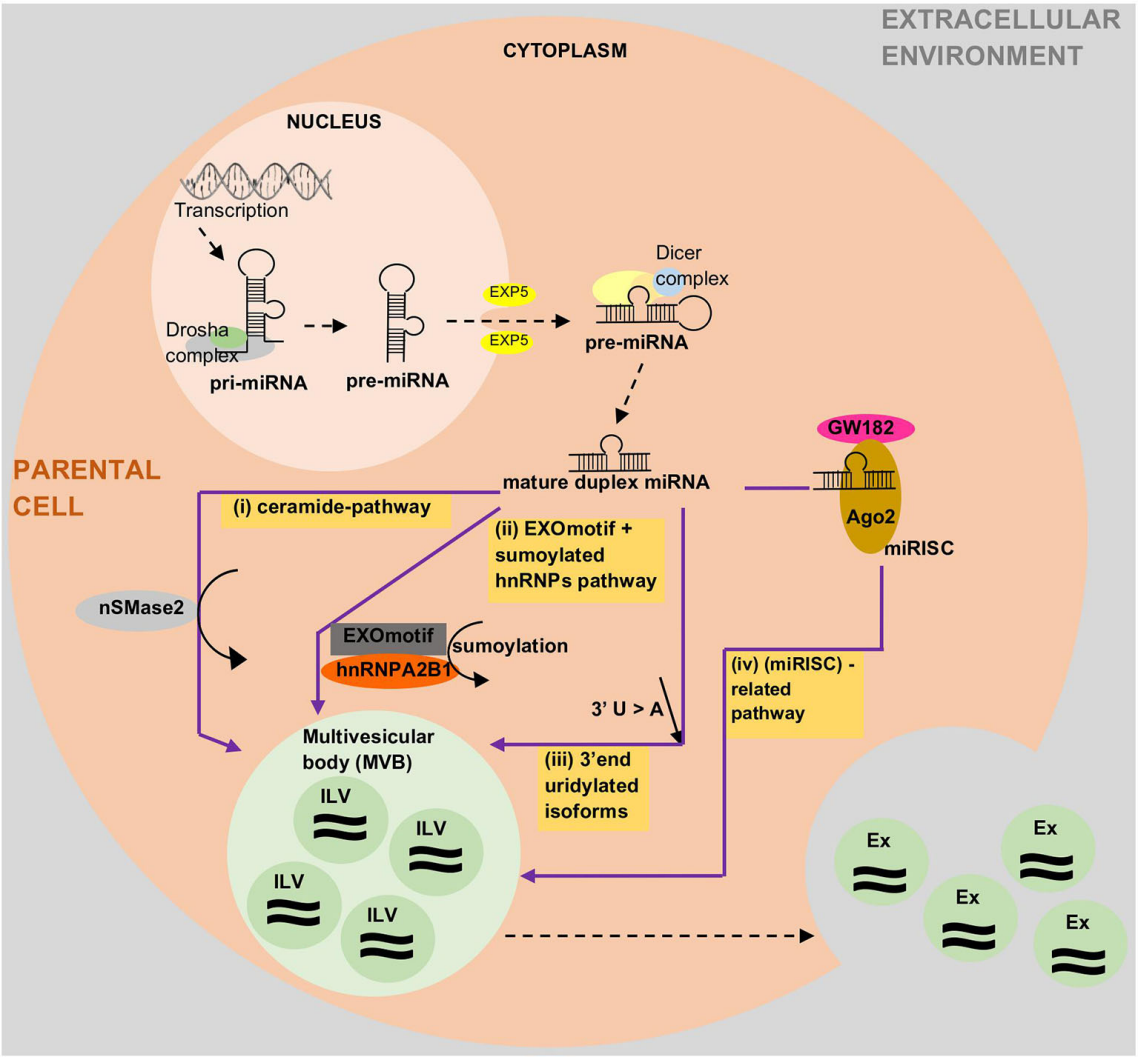
Current state of knowledge on how miRNAs are loaded into exosomes and other extracellular vesicles (EVs). The proposed mechanisms are *via* (i) ceramide pathways, (ii) recognition of EXOmotifs and the sumoylated heterogenous nuclear ribonucleoproteins (hnRNPs)-dependent pathway, (iii) uridylated 3'end isoforms, or (iv) the miRNA-induced silencing complex (miRISC)-related pathway. Ago2, Argonaute-2; Ex, Exosome; EXOmotif, short sequence motifs in miRNAs; EXP5, exportin-5; hnRNPA2B1, heterogenous nuclear ribonucleoprotein A2B1; ILV, intraluminal vesicle; miRISC, miRNA induced silencing complex; nSMase2, neutral sphingomyelinase 2.

Based on the current state of knowledge, there are four potential modes for loading miRNAs into extracellular vesicles (EVs), especially in exosomes, although the underlying mechanisms remain largely unclear (**Figure 2**). The first potential mode is *via* ceramide pathways (Kosaka et al., 2010; Vickers and Remaley, 2012; O'Brien et al., 2018), which are also known as neutral sphingomyelinase 2 (nSMase2)-dependent pathways (Kosaka et al., 2013; Zhang et al., 2015). Sphingolipid ceramide is known to facilitate the process from endosomal vesicle formation to the transportation of miRNAs into exosomes. This ceramide is produced by hydrolysis of sphingomyelin using neutral sphingomyelinases such as nSMase2. Quantitative studies on exosomal miRNAs have shown that the quantity of exosomal miRNAs is directly proportional to the quantity of nSMase2. As such, nSMase2 inhibition by GW4869 resulted in a reduction of exosomes and exosomal-miRNA export. Conversely, an increase in nSMase2 has been shown to increase the number of exosomal miRNAs (Trajkovic et al., 2008; Kosaka et al., 2010; Vickers and Remaley, 2012; Kosaka et al., 2013). The second potential mode is *via* a specific miRNA motif and the sumoylated heterogeneous nuclear ribonucleoprotein (hnRNP)-dependent pathway (Villarroya-Beltri et al., 2013; Zhang et al., 2015; Kim et al., 2017). A study by Villarroya et al. showed two important things that can control the loading of miRNAs into exosomes, namely the short sequence motifs in miRNAs (EXOmotifs) and sumoylation of heterogeneous nuclear ribonucleoprotein A2B1 (hnRNPA2B1). EXOmotifs help hnRNPA2B1 to recognize and bind to specific miRNAs, while sumoylation controls the rate of this binding. Hence, the mutagenesis of EXOmotifs and changes in sumoylated hnRNPA2B1 expression level can affect the loading rate of miRNAs into exosomes. This finding is supported by previous studies that showed the ability of short sequences to guide the transport of RNAs to different subcellular compartments; for example, (1) 20-nucleotide stem-loop sequence was able to direct nuclear-encoded mRNA into mitochondria (Wang et al., 2010), and (2) the hexanucleotide terminal motif of miR-29b was able to direct nuclear enrichment of this miRNA (Hwang et al., 2007; Villarroya-Beltri et al., 2013). The third potential mode is *via* the 3'-end miRNA sequence-dependent pathway (Koppers-Lalic et al., 2014; Zhang et al., 2015). Koppers-Lalic et al. performed a comparison between the 3' end of miRNAs in exosomes and the 3' end of miRNAs in parental cells (B cells). The profiles from RNA sequencing (RNA-seq) showed that 3'-end adenylated miRNAs dominated the parental cells, whereas 3'-end uridylated isoforms dominated the exosomes. A validation study using urine samples confirmed the findings. This suggests that the 3'-end miRNA sequence might play a role in sorting miRNAs into exosomes (Koppers-Lalic et al., 2014). The last potential mode is *via* the miRNA-induced silencing complex (miRISC)-related pathway (Gibbings et al., 2009; Frank et al., 2010; Zhang et al., 2015 and Lee et al., 2009). Mature miRNA can interact with assembly proteins to form a complex called miRISC. miRISC consists of miRNA, miRNA-repressible mRNA, GW182, and AGO2 (Argonaute-2 protein: miRNA effector protein). Any changes in one of the miRISC components may affect the loading of miRNAs into EVs. For example, a crystal structure study on a eukaryotic AGO human protein, AGO2, showed the presence of a middle (MID) domain in the protein. This domain was shown to mediate interaction preferably with adenine (A) and uracil (U) at the phosphorylated 5'-end miRNAs. Thus, these miRNAs will probably be found be to enriched in EVs compared to in miRNAs without U or A at their 5' end (Frank et al., 2010). Other findings supporting the role of miRISC in miRNA sorting into exosomes: (1) the main component of miRISC, GW182, was found to be co-localized in multivesicular bodies (MVBs) and exosomes (Gibbings et al., 2009), (2) blockage of the turnover of MVBs into lysosomes (due to loss of Hermansky-Pudlák Syndrome 4 [HPS4]) could lead to over-accumulation of miRISC and blockage of MVB formation (due to loss of endosomal sorting complex required for transport [ESCRT]), resulting in loss of miRISCs (Lee et al., 2009), and (3) changes in miRNA-repressible target levels caused by cell activation, which leads to sorting of miRNAs into exosomes.

### Description of Exosomes

Exosomes were first discovered by Pan and Johnstone in 1983 during their sheep reticulocyte-related experiment. They termed them ‘a type of small vesicles' responsible for the transportation of receptors into extracellular space during the maturation of sheep reticulocytes (Pan and Johnstone, 1983). Later, Johnstone et al. described these small vesicles visually, using transmission microscopy, and defined the vesicles as exosomes (Johnstone et al., 1989). Exosomes are the smallest vesicles in the EV group, with diameters starting from 0.03 µm and ranging up to 0.1 µm (Chen et al., 2018) or 0.12 µm (Fleissner et al., 2012). The presence of exosomes in a particular source can be confirmed by either: (i) observation of a cup-shaped morphology under negative-staining transmission microscopy or (ii) the presence of exosome markers such as CD63, CD9, and CD81 (Simons and Raposo, 2009; Mathivanan et al., 2010; Gross et al., 2012). Exosomes contain various types of molecules, such as lipids, proteins, lipid rafts, mRNAs, miRNAs, and other non-coding RNAs, such as long non-coding RNAs (lncRNAs) (Zhang et al., 2015). Various cell types can release exosomes, such as reticulocytes (Pan and Johnstone, 1983; Diaz-Varela et al., 2018), dendritic cells (Morelli et al., 2004; Huang et al., 2019), B cells (Benjamin et al., 2019), mast cells (Xie et al., 2018), T cells (Wang et al., 2019), epithelial cells (Lv et al., 2019), and tumor cells (McAtee et al., 2019).

Exosomes are small intraluminal vesicles derived from the exocytosis of multivesicular bodies (MVBs) with plasma membrane (Kosaka et al., 2010; Kim et al., 2017). Specifically, the biogenesis of exosomes starts with the internalization of parental cell membrane to produce endosomes. Small vesicles are formed inside endosomes by invaginating parts of the endosome membranes. These endosomes with small vesicles inside them are called multivesicular bodies (MVBs). MVBs or late endosomes later fuse with parental cell membrane to release the intraluminal endosomal vesicles into the extracellular environment to become exosomes (Zhang et al., 2015). Several molecules have been reported to play crucial roles in the release of exosomes. For example, knockdown of molecules such as Rab27a, Rab27b, or their effectors, SYTL4 and EXPN5, has been shown to result in inhibition of exosome secretion (Ostrowski et al., 2010). Meanwhile, molecules such as tumor repressor protein p53 and its downstream effector, TSAP6, can enhance exosome production (Yu et al., 2006). In addition, the syndecan-syntenin molecule was shown to support the intraluminal budding of endosomal membranes (a crucial step in exosome development) by directly interacting with the ALIX protein (Baietti et al., 2012).

Using exosomes as the source for biomarker development might have several advantages when compared to other existing strategies. The exosome contents reflect the status of parental cells (Momen−Heravi et al., 2015; Pirola et al., 2015; Sohn et al., 2015; Uratani et al., 2016; Liu et al., 2017a; Wang et al., 2017; Yan et al., 2017). Exosomes are also specific (in terms of type and concentration) (Chen et al., 2017; Ebrahimkhani et al., 2017; Perge et al., 2017), which means that they can reflect an individual's health status (Cazzoli et al., 2013). This specificity enables the analysis of low-abundance molecules of interest in a less complex environment (Momen−Heravi et al., 2015; Ramachandran et al., 2017). Exosomes act as a cargo, transferring their biologically active components (Liu et al., 2017a) into targeted cells, playing the role of a cell-to-cell communicator (Pirola et al., 2015; Dinh et al., 2016; Emanueli et al., 2016; Uratani et al., 2016; Weilner et al., 2016; Ramachandran et al., 2017; Fu et al., 2018).

According to the idea that exosomal miRNAs are the best source compared to non-exosomal, cell-free, or whole unfractionated samples, we summarize the selected studies that focus on biomarker findings where the sole source is exosomes in **Table 2**.

### Description of Other EVs

#### Microvesicles (MVs)/ Microparticles (MPs)

Apart from exosomes, the other type of EVs that is recorded by this review analysis as a potential source for circulating miRNAs is microvesicles (MVs)/microparticles (MPs). MVs/MPs range in size from 0.1 to 1 µm in diameter (Chen et al., 2018). These vesicles contain a fraction of the plasma membrane of the parental cells from which the vesicles budded (Kim et al., 2017). Specifically, MVs/MPs are produced *via* direct outward blebbing of parental cell plasma membranes, releasing nascent vesicles into the extracellular environment. Following the blebbing of membrane, distinct and localized changes will occur in the protein and lipid components of the plasma membrane, leading to changes in membrane curvature and rigidity. Proteins that are identified to have functional roles in MV/MP biogenesis are: aminophospholipid translocases (flippasae and floppases) (Stachowiak et al., 2013), ADP-ribosylation factor 1 (ARF1: activation of contractile machinery *via* myosin light chain kinase [MLCK]) (Schlienger et al., 2014), ARF6 (regulates the selective recruitment of proteins into MVs/MPs and activates contractile machinery *via* MLCK) (Muralidharan-Chari et al., 2009), arrestin domain containing 1 (ARRDC1), Tumor susceptibility gene 101 (TSG101) (Nabhan et al., 2012), diaphanous related formin 3 (DIAPH3) (Kim et al., 2014), glutaminase (Li et al., 2012), Hyaluranon synthase (Rilla et al., 2013), localized protein enrichment (Stachowiak et al., 2012), myosin-1a (McConnell et al., 2009), Rab22a (selective recruitment of proteins to MVs/MPs under hypoxic conditions (Wang et al., 2014), and RhoA (links ARF6 activation to MLS phophorylation *via* the ROCK signaling pathway and is involved in actin cytoskeleton rearrangements *via* the RhoA-cofin pathway; Li et al., 2012; Schlienger et al., 2014; Sedgwick et al., 2015). Changes in the above components are complemented by a vertical redistribution of MV/MP cargo components, which are selectively enriched within MVs/MPs (Tricarico et al., 2017).

MVs/MPs can be found in blood circulation (Lacroix et al., 2013; Giannella et al., 2017) as well as other body fluids, such as urine (Smalley et al., 2008), as a result of the activation of cell membrane and/or apoptosis processes (Jesel et al., 2013). MVs/MPs can be shed form various types of cells, such as leukocytes (Pieper et al., 2018), erythrocytes (Yuan et al., 2018), platelets (Diehl et al., 2012; Maugeri et al., 2018), or endothelial cells (Giannella et al., 2017; Zhang et al., 2017). The shed MVs/MPs may or may not contain receptors of their parental cells (Mause et al., 2010; Morel et al., 2011). MVs/MPs are known to participate in biological activities associated with inflammation, immune responses, and thrombosis (Goubran et al., 2015). The intracellular communication of MVs/MPs with targeted cells can be either *via* (1) direct interaction with ligands presented on the surface of targeted cells, (ii) binding of MVs/MPs to the membrane of targeted cells (requires a specific surface antigen), (iii) indirect interaction through modulation of the extracellular environment, or (iv) internalization-fusion to influence gene regulation (Dengler et al., 2013; Goubran et al., 2015; Markiewicz et al., 2013; Mause et al., 2010).

MVs/MPs can be isolated by centrifugation within the range of 13,000 (Zhang et al., 2017) to 20,000 (Jansen et al., 2016) g-force, which can be done without a need for specialized ultracentrifugation machinery. Although this factor seems advantageous toward MVs/MPs as compared to exosomes, the literature shows the opposite, with more studies focusing exosomes than on MVs/MPs. Perhaps, the availability of extensive information regarding exosomes contributes to this phenomenon. Only four studies selected in this review (Diehl et al., 2012; Jansen et al., 2016; Giannella et al., 2017; Zhang et al., 2017) focused solely on MVs/MPs (**Table 2**).

#### Apoptotic Bodies

Apoptotic bodies are the largest vesicles in the EV group, with a diameter range of 1 to 5 µm (Chen et al., 2018). In contrast to exosomes or MVs/MPs, apoptotic bodies are formed only during programmed cell death. The biogenesis of apoptotic bodies starts from apoptosis. A cell that undergoes apoptosis will first experience nuclear chromatin condensation, followed by membrane blebbing and, lastly, the disintegration of cellular components into distinct membranes enclosing vesicles in the apoptotic bodies. Usually, the apoptotic bodies produced will be phagocytosed by macrophages and cleared locally. This clearance process is mediated by interactions between recognition receptors (Annexin V) present on phagocytes with components of the membrane of the apoptotic bodies [phosphatidylserine translocates to the outer leaflet of the lipid bilayer for the interaction (Martinez and Freyssinet, 2001)]. On the other hand, oxidation of surface molecules of apoptotic bodies creates sites for binding of thrombospondin (Savill, 1997; Friedl et al., 2002) or the complement protein C3b (Takizawa et al., 1996). This thrombospondin or C3b can then be recognized by phagocyte receptors (Erwig and Henson, 2008; Mevorach et al., 1998). Hence, Annexin V, thrombospondin, and C3b are used as markers for apoptotic body studies (Savill et al., 1992; Akers et al., 2013).

## Non-Exosomal: Sources Other Than EVs (HDL-Complexes and Ago2-Complexes)

According to the analysis carried out for this review, the source of non-exosomal miRNAs can be either microvesicles (MVs)/microparticles (MPs), as described in the Extracellular Vesicles (EVs) section or from a protein-miRNA complex (such as high-density lipoprotein [HDL] or Argonaute protein [Ago2]) (Redis et al., 2012). In this section, the HDL and Ago2 sources are described.

In general, very few articles report miRNA biomarker studies using solely a non-exosomal source. Only six such articles were successfully retrieved *via* our search strategy, four of which used only microparticles (MPs) miRNAs and two of which used HDL- miRNAs complexes. We summarize the findings in **Table 1**.

### High-Density Lipoprotein (HDL)

High-density lipoprotein (HDL) consists of proteins (75-80%) and lipids (20-25%). The lipids can be fractionated further into phospholipids (65%), triglycerides (30%), and cholesterol (5%). As a major part of HDL is protein, the density of HDL is close to that of protein, which is 1.120 gram per milliliter (Cook and Martin, 1969). The molecular weight of HDL is about 400 kDa, and its diameter ranges from 7 to 20 nm (Burley and Cook, 1961). HDL is formed by a dimer of two monomers, weighing 200 kDa each. Each of the HDL monomers is composed of about five main apoproteins, with molecular weights ranging from 35 to 110 kDa. Each monomer has a funnel-shaped cavity (with a volume of 68 nm^3^), built by 2 beta-sheets (constituted by hydrophobic acids) (Anderson et al., 1998). Each cavity is large enough to fix about 35 molecules of phospholipids concentrated on a monolayer that interact with the cavity's hydrophobic nucleic acid. This hydrophobic cavity protects molecules trapped inside it; as such, phospholipase C can only liberate 6% of the lipid phosphorus of HDLs (Burley and Kushner, 1963; Anton, 2007).

HDL formation is known to taken place in the liver and intestine. The approved biogenesis of HDL involves the interaction of lipid-poor ApoA-1 and ATP binding cassette A1 (ABCA1). ABCA1 is part of the membrane transporter group, functioning as a promoter of transportation of phospholipid and cholesterol from parental cells to poorly lipidated ApoI. The binding of ApoI to ABCA1 has been shown to increase the stability and activity of the transporter in the parental plasma membrane. ATP hydrolysis leads ABCA1 to promote trans-bilayer transportation of phospholipids from the inner to the outer leaflet of the parental plasma membrane. This packing forms extravesiculated lipid domains. ApoI binds to this domain and promotes spontaneous solubilization, which leads to the formation of pre-beta-HDL particles (Canfra´n-Duque et al., 2014; Duong et al., 2006; Vedhachalam et al., 2007). Other proteins and enzymes that are involved in HDL maturation and remodeling include: (i) ATP-binding cassette sub-family G member 1 (ABCG1) (with ABCA1, synergistically mediates cholesterol efflux to HDL) (Gelissen et al., 2006), (ii) scavenger receptor class B type 1 (SRB1) (mediates cholesterol bidirectional flux from cells and HDL, which modulates changes in the composition and structure of HDL particles) (Ji et al., 1997; Jian et al., 1998), (iii) lecithin-cholesterol acyltransferase (LCAT) (mediates fatty acid transportation to pre-beta-HDL to form cholesteryl ester) (Jonas, 1991; Fielding et al., 1972), (iv) cholesteryl ester transfer protein (CETP) (an enzyme that helps in the transportation of both cholesteryl ester and trigylcerides between lipoproteins) (Tall, 1993; Fielding and Fielding, 1995), (v) phospholipid transfer protein (PLTP) (mediates phospholipid transportation from ApoB-containing lipoproteins to HDL), and both (vi) hepatic lipase (HL) and (vii) endothelial lipase (EL) (participate in HDL metabolism) (Connelly, 1999).

Recently, HDL has been reported to act as a transporter in delivering miRNAs to recipient cells, and miRNAs have been proven to influence the gene expression in the recipient cell. For example, in a study where the most abundant miRNA transported by HDL, miR-223 (up to 10,000 copies per microgram of HDL), was transported to Huh7 cells showed a significant reduction of miR-223 target gene expression (Vickers et al., 2011). In addition to that, in this review, two studies (Niculescu et al., 2015; Tabet et al., 2016) involving human samples also shown evidence for miRNA transportation *via* HDL.

Other than miRNAs, HDL is also reported to act as a carrier of fat-soluble vitamins [such as vitamin E (Sattler et al., 1996) and vitamin D (Vaisar et al., 2007; Alwaili et al., 2012)], steroids (lipophilic thyroid hormone thyroxine [T4]; Benvenga et al., 1989), hormones (estrogen, pregnenolone and dehydroepiandrosterone; Tikkanen, et al., 2002), and carotenoids (lycopene, lutein; Krinsky et al., 2004; Vickers and Remaley, 2013).

### Argonaute Protein (Ago2)

The term ‘Argonaute' for proteins comes from the findings of the Bohmert group. The group described a mutant protein in *Arabidopsis thaliana* as AGO1 (Argonaute-1) because the leaf morphology of this plant resembles the shape of a small squid named the ‘greater Argonaut' or *Argonauta argo* (Bohmert et al., 1998). Argonaute proteins can be classified into three paralogous groups, which are: (i) Argonaute-like proteins (resembling *Arabidopsis thaliana* AGO1) (Bohmert et al., 1998), (ii) Piwi-like proteins (closely related to *Drosophila melanogaster* PIWI [P-element induced wimpy testis]) (Lin and Spradling, 1997), and (iii) the *Caenorhabditis elegans-*specific group 3 Argonautes (Yigit et al., 2006).

There are four domains in an Argonaute protein, namely the N-terminal, PAZ (Piwi-Argonaute-Zwille), Mid (Middle), and PIWI (Parker et al., 2005). The N-terminal domain is involved in (i) the loading of small RNAs to the protein, (ii) assisting in the unwinding process of small RNA duplexes (Kwak and Tomari, 2012), and (ii) together with the PIWI domain, its unstructured loop is plays an important role in the cleavage process (Hauptmann et al., 2013). The PAZ domain is responsible for recognizing the 3'-overhangs of small RNAs and leads the small RNAs into a specific binding pocket inside the protein (Jinek and Doudna, 2009). The MID domain provides a binding pocket at which the 5'-terminal bases of small RNAs can form stacking interactions with a conserved tyrosine within it. In addition, several hydrogen bonds help to coordinate correct 5'-end binding. The PIWI domain is responsible for endonucleases, which cleave target RNA that is fully complementary to the bound small RNA (Jinek and Doudna, 2009). However, not all Argonaute proteins possess cleavage activity. For example, in mammals, only AGO2 of the AGO subfamily is catalytically active and functioning as an endonuclease (Liu et al., 2004; Meister et al., 2004).

Apart from being a cleavage location for bound miRNA target genes, Argonaute proteins also play a role as miRNA carriers. This carrier role has been demonstrated in several articles that have been analyzed in this review, for example: (i) Arroyo et al. found that 90% of miRNAs in circulation (from human plasma samples) were present in a non-membrane-bound form consistent with a ribonucleoprotein complex (Ago2 ribonucleoprotein complex) and that this complex provides stability for nonvesicular circulating miRNAs (Arroyo et al., 2011); (ii) the Turchinovich et al. study showed similar results to that of Arroyo et al.; that is, that the majority of nuclease-resistant extracellular miRNAs in both plasma and cell culture media is floating outside exosomes and is bound to the Ago2 protein (Turchinovich et al., 2011).

## Performance Comparison of Exosomal and Non-Exosomal Circulating mirnas as Potential Biomarkers

**Tables 3** and **4** summarize the articles on the role and mechanism of circulating miRNAs and their potential to be used as biomarkers. Out of the total of 69 articles cited, six were exclusively on non-exosomes, 31 were exclusively on exosomes, and the remaining 32 related to both exosomes and non-exosomes. Out of these 32, 18 articles pointed to exosomal miRNAs, six to non-exosomal miRNAs, and 7 to unfractionated samples as a better source for miRNAs used as a biomarker. However, one article differed from the above, stating that similar expression was observed both in whole and exosomal serum samples (**Figure 1**).

Based on the above data, it is obvious that 71% of the articles (31 + 18) concluded that exosomes are the source of choice for miRNAs in biomarker studies. Moreover, taking into consideration the articles (18) that compared both exosomal and non-exosomal miRNAs, 75% recommended an exosomal source of miRNAs over non-exosomal miRNAs. Though the debate on the preference and recommendation of the source of miRNAs, whether exosomal or non-exosomal, is never-ending, summing up the information based on the literature, it can be inferred that the exosomal source of miRNAs can act as a better source for biomarker studies owing to its advantages in terms of quantity, quality, and stability. Hence, the exosomal source of miRNAs holds promise in studies related to biomarkers and diseases, which can be reaped for the benefit of mankind in terms of screening and surveillance. Having said that, the role of other biomarkers for disease diagnosis from other sources cannot be ignored altogether. Further exploration is needed to ascertain how other biomarkers can be used hand-in-hand with miRNAs from exosomal sources; these may complement each other and make disease diagnosis more efficient and timely. This hence warrants further research, exploring other possible resources that can be reaped for early diagnosis, prevention, and treatment of diseases.

## Significance Statement

This review was written with the aim to compare all studies pertaining to circulatory miRNA and to evaluate the validity of using exosomal or non-exosomal as biomarker in diseases.

## Author Contributions

NN and WS wrote or contributed to the writing of the manuscript.

## Funding

This review is part of research funded by the Fundamental Research Grant Scheme (FRGS), Malaysia [Grant number: FRGS/1/2016/203.PPSG. 6171194].

## Conflict of Interest

The authors declare that the research was conducted in the absence of any commercial or financial relationships that could be construed as a potential conflict of interest.

## Abbreviations

ACA, adrenocortical adenomas; ACC, adrenocortical cancer; Ago, Argonaute; ALP, alkaline phosphate; AMI, acute myocardial infarction; ART, anti-retroviral therapies; AUC, area under the curve; CA, cancer antigen; CAD, coronary artery disease; CEA, carcinoembryonic antigen; CRC, colorectal cancer; CRPC, castration-resistant prostate cancer; CT, computed tomography; cTnI, cardiac troponin I; ESCC, esophageal squamous cell carcinoma; EMP, endothelial microparticle; FSGS, focal segmental glomerulosclerosis; HDL, high-density lipoprotein; HCC, hepatocellular carcinoma; HF, heart failure; HP, high protein; hsa, “*homo sapiens*” = human microRNA prefix; IH, intermittent hypoxia; let, microRNA prefix that is conserved in many species; LT, liver transplant; OHRQoL, oral health-related quality of life; OvCa, ovarian cancer; MCD, minimal change disease; miRNA, microRNA; miR, mature form of microRNA, MoCA, Montreal Cognitive Assessment; MP, microparticle; MS, multiple sclerosis; MRI, magnetic resonance imaging; MVB, multivescular bodies; NAFLD, non-alcoholic fatty lipid disease; NPC, nasopharyngeal carcinoma; NSCLC, non-small cell lung cancer; PEG, polyethylene glycol; PET, positron emission tomography; PCa, prostate cancer; qPCR, quantitative polymerase chain reaction; RISC, RNA-induced silencing complex; ROC, receiver operating curve; RNP, ribonucleoprotein; RT-PCR, reverse transcription polymerase chain reaction; RT-qPCR, real-time reverse transcription polymerase chain reaction; SEC, size-exclusion chromatography; SCAD, stable coronary artery disease; SCC, squamous cell cancer; SS, synovial sarcoma; TNM, tumor/nodes/metastasis; UA, unstable vagina; UC, ultracentifugation; UM, uveal meloma; VH, citreous humor; VHR, very high risk.
